# Chromosomal Roadblocks in Male Fertility: Mechanisms, Risk Factors and Syndromes

**DOI:** 10.3390/medicina61101864

**Published:** 2025-10-16

**Authors:** Achilleas G. Mitrakas, Christina-Angelika Alexiadi, Sofia Gargani, Triantafyllos Alexiadis, Sofia-Panagiota Alexopoulou, Olga Pagonopoulou, Maria Lambropoulou

**Affiliations:** 1 Laboratory of Histology-Embryology, Medical School, Faculty of Health Sciences, Democritus University of Thrace, 68100 Alexandroupolis, Greecealexiadistr@gmail.com (T.A.); sof.alexopoulou@gmail.com (S.-P.A.);; 2 Department of Histology and Embryology, Medical School, Faculty of Health Sciences, Aristotle University of Thessaloniki, 54124 Thessaloniki, Greece; 3 Laboratory of Neurophysiology, Medical School, Faculty of Health Sciences, Democritus University of Thrace, 68100 Alexandroupolis, Greece; opagonop@med.duth.gr

**Keywords:** male fertility, chromosomal abnormalities, infertility mechanism, spermatogenesis, Y chromosome microdeletion, azoospermia, oligospermia

## Abstract

Male infertility affects nearly 15% of couples worldwide, with chromosomal abnormalities representing a major underlying cause. This review explores how numerical and structural chromosomal anomalies, along with environmental exposures, lifestyle factors, and age-related genetic changes, disrupt spermatogenesis and contribute to infertility. It synthesizes findings from cytogenetic, molecular, and clinical studies, with particular focus on mechanisms such as meiotic nondisjunction, spindle assembly checkpoint dysfunction, and alterations in cohesin and synaptonemal complex proteins. Chromosomal abnormalities, both numerical and structural, emerge as key contributors to male infertility by impairing chromosomal segregation and recombination, often leading to azoospermia or oligospermia. Meiotic checkpoint failures and recombination errors further exacerbate the production of aneuploid sperm. Environmental toxins, oxidative stress, and poor nutrition disrupt hormonal balance and chromatin integrity, while advancing paternal age is associated with increased sperm aneuploidy and impaired meiotic control, with implications for assisted reproduction. Specific syndromes, including AZF deletions, Kallmann syndrome, and 46,XX testicular DSD, exemplify the direct genetic impact on male fertility. Overall, chromosomal abnormalities are central to the pathophysiology of male infertility, arising from intrinsic meiotic errors as well as extrinsic environmental and lifestyle factors. Integrating cytogenetic diagnostics, genetic counseling, and lifestyle interventions is essential for comprehensive fertility assessment and management. Further research into molecular biomarkers and targeted therapies could enhance diagnosis, improve treatment strategies, and lead to better reproductive outcomes.

## 1. Introduction

Genetic disorders are, traditionally, classified into three main categories: (i) single-gene defects, (ii) chromosomal abnormalities, and (iii) multifactorial conditions. Among these, the chromosomal abnormalities- the structural or numerical alterations in one or more chromosomes- are crucial contributors for a wide range of clinical outcomes [1,2]. The human genome is organized into 46 chromosomes (22 pairs of autosomes and one pair of sex chromosomes, 44+XX in females, 44+XY in males), collectively harboring approximately 20,000 to 25,000 genes vital for the development, growth and cellular functions. Errors during cell division, either mitosis or meiosis, can lead to chromosomal abnormalities with significant clinical consequences, such as spontaneous abortion, stillbirth, congenital malformations, intellectual disability, and recognizable syndromic presentations [3]. Early and accurate detection of these abnormalities is crucial for effective genetic counseling, informed reproductive decision-making, and the implementation of targeted interventions [4]. Infertility, a condition affecting nearly 15% of couples worldwide, is influenced by male factors in approximately half of all cases [5]. Among the leading causes of male infertility are genetic and chromosomal abnormalities, particularly the production of aneuploid sperm, gametes with an abnormal number of chromosomes [6]. Such abnormalities can impair fertilization, lead to early embryonic loss, or result in live births with congenital anomalies. A comprehensive understanding of the mechanisms underlying chromosomal instability in male gametogenesis is therefore essential not only for diagnosis and treatment of infertility but also for the development of prevention strategies and effective genetic counseling [7].

## 2. Types of Chromosomal Abnormalities

### 2.1. Numerical Abnormalities and Association with Infertility

Numerical abnormalities are a type of chromosomal disorder characterized by an atypical number of chromosomes within a cell. Normally, human cells contain 46 chromosomes arranged in 23 pairs; however, numerical abnormalities occur when one or more chromosomes are either missing or present in excess. These anomalies typically arise during cell division—particularly meiosis—due to errors in chromosome segregation. Common examples include trisomies, where an individual has three copies of a particular chromosome (e.g., trisomy 21, which causes Down syndrome), and monosomies, where one chromosome is missing. In the context of reproductive health, numerical chromosomal abnormalities in germ cells can result in infertility, recurrent miscarriages, or congenital disorders in offspring [8].

Klinefelter syndrome (47,XXY) is the most frequently observed numerical chromosomal abnormality in infertile males, present in almost 0.1–0.2% of newborn males and up to 3–4% of infertile men. Moreover, there are other numerical abnormalities including 47,XYY syndrome (about 1 out of 1000 male children) [9], 45,X/46,XY mosaicism (5.6 and 2.1 per 100,000 liveborn males and females, respectively) [10], and various forms of aneuploidy in somatic or germ cells. Studies using sperm FISH (fluorescence in situ hybridization) techniques have demonstrated increased disomy and nullisomy rates in men with these conditions [11].

Klinefelter syndrome is characterized by the presence of one extra X chromosome in males, which leads to impaired testicular function, reduced testosterone levels, and azoospermia or severe oligozoospermia in most affected individuals. In certain cases, individuals may carry more than one additional X chromosome yet display a phenotype similar to that of XXY Klinefelter syndrome. Although some mosaic variants (such as 46,XY/47,XXY) may preserve a limited capacity for fertility, most non-mosaic cases are typically identified during infertility assessments [12]. In addition to hormonal imbalances, increased rates of sex chromosome aneuploidy in sperm have been documented in these patients, which may raise the risk of chromosomal abnormalities in offspring conceived through assisted reproductive techniques [13].

Although less commonly associated with infertility, other numerical abnormalities—such as 47,XYY syndrome (Jacob’s syndrome)—can still affect spermatogenesis in a subset of affected individuals [14]. Another important example of a numerical chromosomal abnormality affecting fertility is Turner syndrome, which is usually marked by the presence of a single X chromosome (45,XO) instead of two sex chromosomes. XO embryos account for about 3% of all conceptions, the vast majority of which—around 99%—result in spontaneous pregnancy loss within the first trimester [15]. Turner syndrome, which affects phenotypic females, is characterized by primary ovarian insufficiency, short stature, and other congenital anomalies. While individuals with classic Turner syndrome (45,XO) are usually infertile due to gonadal dysgenesis, certain mosaic forms (such as 45,XO/46,XX or 45,XO/46,XY) may have spontaneous pubertal development and, in rare cases, even fertility potential [16]. However, pregnancies in women with Turner syndrome, especially those achieved through assisted reproductive technologies, carry significant risks and require careful medical supervision [17].

The 45,XO/46,XY mosaicism is another complex condition that is often linked with disorders of sex development (DSDs). Individuals with this mosaicism can present a wide range of phenotypes, from apparently normal male or female genitalia to varying degrees of genital ambiguity. These individuals generally have reduced fertility, and there is an elevated risk of developing gonadal tumors, especially gonadoblastoma [18]. Early diagnosis allows the decisions regarding management, fertility preservation options, and appropriate genetic counseling [19].

### 2.2. Structural Abnormalities and Association with Infertility

Structural chromosomal abnormalities are defined as alterations in the structure of the chromosomes, including various types of alternations, such as translocations, inversions, deletions, and duplications. These changes can disrupt normal genetic function, gene dosage, or the alignment and segregation of chromosomes during meiosis. Among the most significant contributors to infertility are Robertsonian translocations (around 1 per 800 individuals) [20], which involve the fusion of two acrocentric chromosomes, and reciprocal translocations, where chromosome segments are exchanged between nonhomologous chromosomes [21]. These rearrangements often result in the formation of unbalanced gametes, leading to reduced fertility, miscarriage, or offspring with chromosomal disorders [22,23].

In males, structural abnormalities are a well-established cause of infertility, primarily due to their impact on spermatogenesis [22]. Microdeletions on the Y chromosome [13], especially within the AZF regions, represent one of the leading genetic causes of severe oligospermia or azoospermia (prevalence range from less than 2% to over 24% based on ethnicity and geographical region) [24,25]. Deletions result in the loss of essential genes required for spermatogenesis. Pericentric inversions, such as chromosome 9 rearrangement (1–3% in the general population while is around 0.25% among Asians) [26,27], are also frequently found, although their clinical significance remains controversial; some consider them benign variants [28], while others suggest a possible link to reduced fertility or miscarriage depending on the size and position of the inversion [29].

From an epidemiological perspective, structural abnormalities are more commonly associated with male infertility than female [30]. In females, while such chromosomal rearrangements can affect fertility, they more frequently result in recurrent pregnancy loss or embryonic aneuploidy, rather than primary infertility [31]. The clinical outcome of these abnormalities largely depends on the chromosomes involved, the specific genes affected, and the size of the rearranged segments. Genetic counseling and cytogenetic analysis are essential tools in evaluating couples with unexplained infertility, helping to identify underlying chromosomal factors and guide appropriate reproductive options (Table 1).

While some authors argue that inv(9) is a benign variant, others report associations with impaired spermatogenesis, suggesting a possible context-dependent pathogenicity. This discrepancy highlights the need for high-resolution molecular studies to determine whether specific breakpoint locations or epigenetic alterations are responsible for the observed fertility effects.

## 3. Mechanisms of Aneuploidy in Spermatogenesis

### 3.1. Meiotic Nondisjunction

Aneuploidy is the presence of an abnormal number of chromosomes, is a significant cause of male infertility, miscarriages, and congenital disorders such as Down syndrome. One of the primary mechanisms leading to aneuploidy in spermatogenesis is meiotic nondisjunction. This describes the faulty separation of chromosomes during meiosis, the specialized form of cell division that produces haploid sperm cells from diploid precursors. Nondisjunction can occur during either meiosis I or II, resulting in sperm that lack or carry extra chromosomes [32].

While much attention has historically focused on maternal contributions to aneuploidy, increasing information underscores that paternal nondisjunction plays a pivotal role, particularly in cases involving sex chromosomes [33,34,35]. The male germline generally exhibits a lower rate of aneuploidy than the female counterpart, possibly due to stricter meiotic checkpoints and the selective apoptosis of abnormal spermatocytes.

During meiosis I, homologous chromosomes are meant to pair, undergo recombination (crossing over), and segregate into separate cells. Nondisjunction at this stage typically results in one cell receiving both homologs while the other receives none [36]. This is often due to errors in synapsis or recombination, especially when chiasmata fail to form correctly between homologous chromosomes. It is possible chromosomes may not align correctly on the meiotic spindle, without proper recombination, leading to missegregation. Spermatocytes that do not undergo at least one crossover per chromosome pair are at especially high risk of nondisjunction [37]. This is often referred to as the “obligate crossover” requirement, a procedure that has been studied in a variety of organism [38], and its failure is a key contributor to meiotic error [36,39].

**Figure 1:**
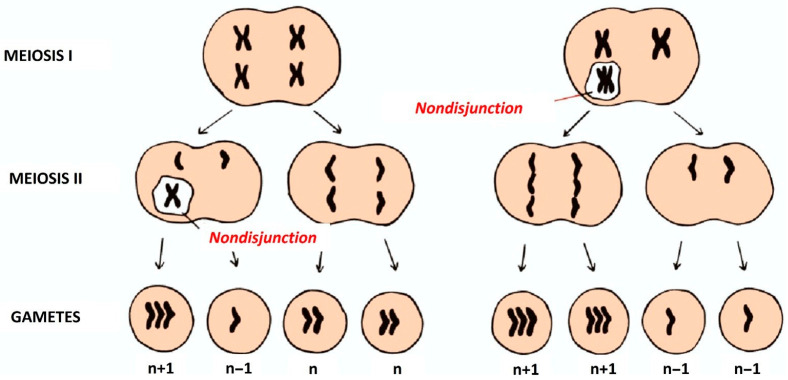
Aneuploid gametes arising from meiotic nondisjunction are a major cause of male infertility.

Meiosis II presents similarities with mitotic division, wherein the sister chromatids of each chromosome are supposed to separate. Nondisjunction in meiosis II results in one daughter cell inheriting both sister chromatids, while the other inherits none. This may be due to a variety of reasons, such as spindle assembly checkpoint defects, cohesion failure, or improper kinetochore attachment. In contrast, nondisjunction during meiosis I is associated with unbalanced homologous chromosomes and defective recombination, meiosis II nondisjunction typically arises from defects in chromatid cohesion, which is supposed to hold sister chromatids together until anaphase II (Figure 1) [40].

As already referred, while nondisjunction is more commonly associated with oogenesis, growing evidence indicates it also occurs in spermatogenesis. However, the incidence of aneuploid sperm is typically lower than that of aneuploid oocytes. Nonetheless, age-related increases in sperm aneuploidy have been documented, particularly for sex chromosomes, suggesting that the fidelity of chromosome segregation declines with paternal aging [32,40].

In conclusion, meiotic nondisjunction is a key mechanism contributing to aneuploidy in spermatogenesis. It can result from errors in recombination, chromatid cohesion, or spindle function, and its consequences range from reduced fertility to the transmission of chromosomal disorders. A thorough understanding of these mechanisms is essential for diagnosing and potentially treating male infertility, as well as for preventing conceptions involving chromosomal abnormalities.

### 3.2. Cohesin and Synaptonemal Complex Defects

Cohesin and synaptonemal complex (SC) defects are crucial contributors to meiotic failure, leading to aneuploidy during spermatogenesis, with profound implications for male fertility [41]. The cohesin complex comprises a ring-shaped group of proteins, primarily Structural Maintenance of Chromosomes protein 1 (SMC1), Structural Maintenance of Chromosomes protein 3 (SMC3), REC8 Meiotic Recombination Protein, and cohesin subunit SA-3 (STAG3), that encircle sister chromatids, holding them together from DNA replication in premeiotic S-phase until their separation during meiotic division [36,37,38,39]. STAG3 is the meiosis-specific subunit of the cohesin complex, which replaces the mitotic STAG1/2 isoforms and plays a pivotal role in stabilizing chromatid cohesion and homologous chromosome pairing during prophase I [40,41]. STAG3 interacts with SMC1β, SMC3, and REC8 to form the core ring structure that encircles sister chromatids. Variants in cohesin components, such as *SMC1* [42], or the existence of specific variants, like *STAG3* [41,43], have been associated with impaired homolog pairing, premature chromatid separation, and meiotic arrest, which can result in oligozoospermia or azoospermia. In *Stag3−/−* mouse models, spermatocytes experience a complete meiotic arrest at the zygotene stage, accompanied by severe synapsis failure, disrupted axis formation, and persistent double-strand DNA breaks. These findings confirm that STAG3 is crucial for the assembly of the axial elements of the synaptonemal complex and for crossover formation. Furthermore, recent studies in humans have identified biallelic pathogenic variants in STAG3 in men with non-obstructive azoospermia, underscoring its clinical importance as a key factor in meiotic failure and infertility [44].

Simultaneously, the synaptonemal complex (SC) is protein scaffold involving three separate parts. This scaffold forms between homologous chromosomes during prophase I, enabling synapsis and promoting crossover formation. The SC is composed of lateral elements [(e.g., Synaptonemal Complex Protein 2 (SYCP2) and Synaptonemal Complex Protein 3 (SYCP3)] [45], a central element [Synaptonemal Complex Central Element Protein 1–3 (SYCE1–3), Testis Expressed 12 (TEX12)] [34], and the transverse filament protein SYCP1 [46]. In different species is exhibited that SYCP1 is significant fir chromosome synapsis [47]. Proper SC formation has a pivotal role for stabilizing homolog pairing and ensuring successful recombination [48]. Deficiencies in SC proteins can result in unsynapsed chromosomes, defective crossover events, and persistent DNA double-strand breaks [49]. For instance, variants in *SYCP3* have been linked to male infertility due to meiotic arrest and apoptosis of spermatocytes. SYCP3, a component of the lateral element of the synaptonemal complex (SC), plays a key role in the alignment and synapsis of homologous chromosomes. SYCP3 polymerizes along the axial cores and is essential for SC stability. It forms a heterodimer with SYCP2, anchoring the complex to cohesin-loaded chromatin axes. *Sycp3*−/− knockout mice display defective axial element assembly, synapsis failure, and extensive apoptosis of spermatocytes, confirming its indispensability for meiotic progression [50]. In humans, mutations in *SYCP3* have been identified in men with azoospermia or cryptozoospermia [51], often associated with complete meiotic arrest [52]. Structural variants lead to truncated proteins unable to dimerize or bind chromatin, underlining the importance of SYCP3’s α-helical domain in SC formation [51,53]. In humans, SC or cohesin abnormalities can be identified in infertile men, particularly those with azoospermia. Moreover, specific miRNAs are associated with this system influencing normal spermatogenesis (Table 2) [54].

### 3.3. Clinical and Translational Implications of Cohesin and Synaptonemal Complex Defects

Recent findings underscore that cohesin and synaptonemal complex (SC) dysfunction extends beyond meiotic arrest, with important implications for male reproductive health. Age-related weakening of chromatid cohesion has been linked to increasing rates of sperm aneuploidy, mirroring observations in oocytes and highlighting a potential mechanism contributing to paternal age effects [40,57,58,59,60,61]. In addition, epigenetic regulators and non-coding RNAs have been shown to influence the expression of STAG3 and SYCP3, placing cohesin- and SC-mediated pathways at the crossroads of genetic and environmental interactions [41,43,51]. Clinically, pathogenic variants in cohesin and SC genes are increasingly identified through next-generation sequencing panels in infertile men, providing valuable diagnostic and prognostic information, especially in cases of non-obstructive azoospermia. Looking ahead, novel CRISPR-based models and high-resolution imaging approaches offer promising tools for dissecting cohesin- and SC-related mechanisms in spermatogenesis and may eventually pave the way for targeted therapeutic interventions [41,43,51].

### 3.4. Spindle Assembly Checkpoint Dysfunction

The spindle assembly checkpoint (SAC) is a critical surveillance mechanism that ensures the accurate segregation of chromosomes during meiosis by delaying anaphase onset until all chromosomes are properly attached to the spindle microtubules [51,52]. In spermatogenesis, where continuous production of sperm relies on tightly regulated meiotic divisions, the SAC plays a pivotal role, preventing premature progression through meiosis in the presence of kinetochore-microtubule attachment errors [62]. There are more than fourteen (14) proteins that participate in the SAC, such as Mitotic Arrest Deficient 1 (MAD1), Mitotic Arrest Deficient 2 (MAD2), Budding Uninhibited by Benzimidazole-Related 1 (BUBR1) Budding Uninhibited by Benzimidazole 1 (BUB1) and Budding Uninhibited by Benzimidazole-Related 3 (BUB3) and Cell Division Cycle 20 (CDC20) [63], inhibiting the anaphase-promoting complex/cyclosome (APC/C) (with ubiquitin ligase activity) until correct bipolar attachment is achieved for every chromosome [64]. Moreover, the significant role of SAC in meiosis II has proved, as well [65]. When the SAC is impaired, either through genetic mutations, abnormal expression, or age-related deterioration, chromosomes can missegregate, resulting in sperm with an incorrect number of chromosomes [66,67]. These numerical chromosomal abnormalities, or aneuploidies, are a leading cause of male infertility, embryonic lethality, and congenital syndromes when such sperm fertilize a normal egg.

**Figure 2:**
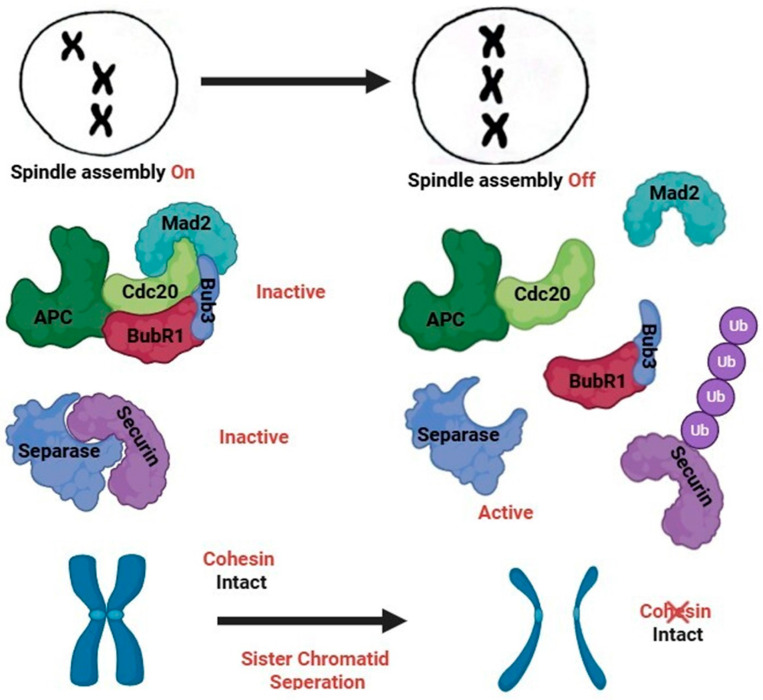
Spindle Assembly Checkpoint Dysfunction. The Anaphase-Promoting Complex/Cyclosome (APC/C) is a multi-subunit E3 ubiquitin ligase that regulates progression through mitosis and meiosis. Once proper chromosome alignment is achieved, APC/C targets securin and cyclins for degradation, triggering sister chromatid separation and anaphase onset. The SAC (Spindle Assembly Checkpoint) inhibits APC/C via CDC20 until all chromosomes are correctly attached to the spindle, ensuring accurate chromosome segregation and genomic stability.

Defective SAC function in male germ cells has been linked to both meiotic arrest and the production of aneuploid gametes, as well as gametes with structural abnormalities such as Robertsonian translocations [67]. In some cases, failure to activate the checkpoint properly allows cells with misaligned or unpaired chromosomes to proceed through meiosis, leading to disomic or nullisomic sperm. In other instances, prolonged SAC activation caused by unresolved chromosome-spindle attachment errors can trigger apoptosis of spermatocytes, contributing to low sperm counts or azoospermia [68]. Studies in experimental models, including mice with targeted deletion or dysfunction of SAC genes like *Bub1b* or *Mad2*, demonstrate that SAC failure during spermatogenesis leads to widespread chromosome missegregation, reducing fertility [69]. Additionally, oxidative stress, environmental toxins, and advanced paternal age have been linked to impaired SAC signaling, suggesting that both intrinsic and extrinsic factors can influence checkpoint robustness [70,71]. Understanding SAC dysfunction provides critical insight into the origin of sperm aneuploidy and highlights potential diagnostic or therapeutic targets for improving male reproductive health (Figure 2) [72].

## 4. Influence of Paternal Age

Advanced paternal age has already recognized as a possible contributing factor to chromosomal abnormalities and the formation of aneuploid sperm, challenging the long-standing notion that maternal age is the primary driver of gametic aneuploidy [57]. Although spermatogenesis continues, almost, throughout a man’s life, the continuous mitotic divisions of spermatogonia lead to the gradual accumulation of replication errors, DNA damage, and epigenetic alterations [58,59]. These factors contribute to genomic instability in older males, which may affect chromosome segregation during meiosis [59]. Advanced paternal age has been associated with reduced fertility [60], which in turn could affect the success rate of in vitro fertilization. As mentioned above, one key mechanism is the age-related weakening of meiotic checkpoint surveillance and cohesion between sister chromatids, which can increase the risk of nondisjunction during meiotic divisions. Particularly, the incidence of disomy for chromosomes X, Y, 13, 18, and 21 has been observed to rise with paternal age, potentially resulting in conditions such as Klinefelter syndrome (47,XXY), Turner syndrome (45,X), and Down syndrome (trisomy 21), when fertilization occurs. This challenges the long-standing notion that maternal age is the sole or primary driver of gametic aneuploidy, highlighting that advancing paternal age also contributes significantly to chromosomal abnormalities in male gametes [61]. It is suggested that while telomerase activity remains consistently high in spermatogonia throughout a male’s life, oocytes and early embryonic stages exhibit little to no detectable telomerase expression [73].

Molecular and cytogenetic studies have confirmed that sperm from older men carry a higher rate of structural and numerical chromosomal abnormalities [74]. Fluorescence In situ Hybridization (FISH) analyses have demonstrated that men over 40 years of age exhibit significantly higher rates of sex chromosome and autosomal disomies compared to younger counterparts [74]. Furthermore, oxidative stress in aging testicular tissue can cause DNA strand breaks and impair chromatin remodeling during spermiogenesis, exacerbating the risk of abnormal sperm formation [75]. Although most aneuploid sperm do not result in successful fertilization due to natural selection or embryonic arrest, the use of assisted reproductive technologies, such as Intracytoplasmic Sperm Injection (ICSI) [76], may bypass these selection barriers and increase the likelihood of passing chromosomal errors to the offspring [76]. Given these risks, paternal age should be considered in genetic counseling and reproductive planning, particularly in couples considering delayed parenthood.

## 5. Environmental and Lifestyle Factors

### 5.1. Introduction

**Figure 3:**
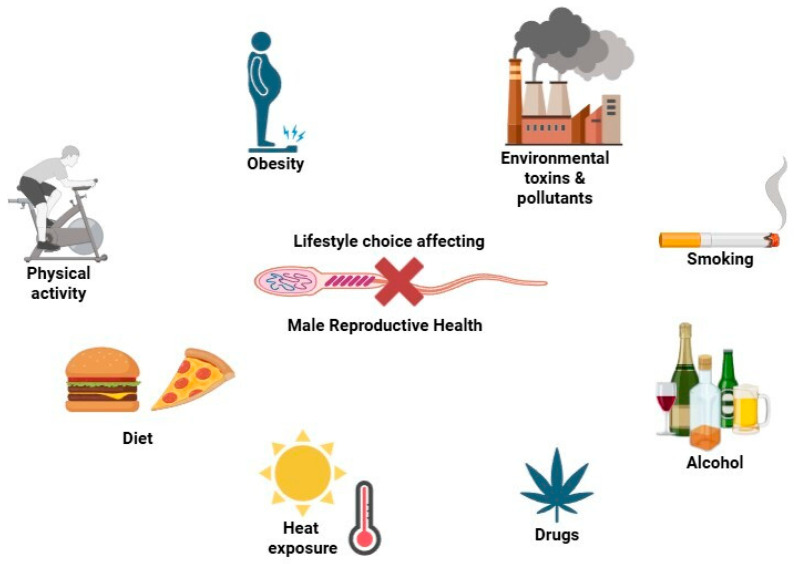
Environmental and lifestyle factors—such as toxins, poor diet, smoking, and stress—can disrupt spermatogenesis by interfering with hormonal regulation, meiosis, and chromosomal integrity. These modifiable influences are key contributors to reduced sperm quality and increased risk of aneuploidy, highlighting their relevance in male fertility and reproductive health.

Environmental and lifestyle factors play a vital role in male reproductive health, especially in spermatogenesis and maintaining chromosomal stability. Because these external and behavioral influences are modifiable, they represent important targets for fertility preservation. Growing evidence shows that exposure to environmental toxins, unhealthy habits, and chronic stress can impair sperm quality, disrupt meiosis, and elevate the risk of chromosomal abnormalities like aneuploidy (Figure 3).

### 5.2. Environmental Toxins and Pollutants

Exposure to environmental pollutants, such as pesticides, heavy metals (lead, cadmium) [77], microplastics and plasticizers (phthalates) [78], and air pollution, that has been associated with oxidative stress [79], DNA damage, and impaired meiotic processes. These substances can interfere with the endocrine system and disrupt hormonal signaling critical for normal spermatogenesis [80]. For instance, occupational exposure to industrial chemicals has been linked to increased sperm DNA fragmentation and higher frequencies of disomy, particularly in chromosomes 13, 18, 21, X, and Y. Long-term exposure can also affect epigenetic programming [81], leading to transgenerational reproductive effects [82].

Despite clear associations between toxic exposures and sperm aneuploidy, current evidence is limited by inconsistencies in exposure measurement and individual susceptibility. Future studies should aim to stratify patients by genetic background to better understand inter-individual variability in toxin response.

### 5.3. Lifestyle Factors: Smoking, Alcohol, and Drugs

Tobacco smoking is strongly correlated with increased sperm DNA damage and oxidative stress [83,84]. Oxidative stress can contribute to chromosomal missegregation during meiosis, leading to infertility [85]. The use of recreational drugs (cannabis, anabolic steroids) effect on testicular morphology, meiotic progression, and sperm chromatin condensation [86,87]. Likewise, excessive alcohol consumption can alter testosterone levels [88] and impair the hypothalamic-pituitary-gonadal axis [89], reducing spermatogenic efficiency and increasing the risk of producing chromosomally abnormal sperm. While advising patients with fertility concerns to avoid smoking, alcohol, and recreational drugs is sound health guidance, strong scientific evidence directly linking recreational drug use to impaired sperm production remains limited at present.

### 5.4. Diet and Nutrition

Diet and nutrition play a significant role in regulating spermatogenesis [90]. Adequate nutrient intake maintains testicular function and hormonal balance, whereas deficiencies are linked to oxidative stress, DNA damage, and spindle dysfunction, thereby increasing sperm aneuploidy and the risk of infertility, miscarriage, and chromosomal disorders such as Down syndrome or Klinefelter syndrome [91]. Optimizing diet is therefore a key preventive strategy [92].Vitamins play pivotal roles: vitamin A supports the blood–testis barrier [93], vitamin C improves sperm count, motility, and morphology [94] and vitamin E enhances assisted reproduction outcomes [95], with low levels noted in oligospermia and asthenozoospermia [96]. Vitamin D also regulates sperm maturation, with positive correlations reported between serum levels and motility [97], potentially reducing chromosomal abnormalities [98]. Together, these findings underscore the protective role of antioxidants against oxidative stress in spermatogenesis.

Beyond vitamins, essential micronutrients further safeguard sperm integrity. Selenium neutralizes ROS, with deficiencies causing impaired motility and reduced fertility, while zinc, as a cofactor in transcription and protein synthesis, improves sperm morphology, motility, and semen volume, often reduced in infertile men [99]. Similarly, coenzyme Q10 has been associated with higher sperm density, motility, and the proportion of morphologically normal sperm [100,101]. Collectively, these dietary and micronutrient factors highlight nutrition as a modifiable determinant of chromosomal stability and male reproductive potential.

### 5.5. Physical Activity, Heat Exposure, and Obesity

Engaging in regular, moderate physical activity plays a supportive role in maintaining hormonal balance and managing oxidative stress—two key factors for healthy sperm development. When performed consistently and in balance with proper recovery and nutrition, exercise can promote optimal conditions for spermatogenesis [102,103]. However, excessive or intense physical training, particularly when coupled with inadequate recovery or nutritional deficits, can lead to hormonal imbalances such as reduced testosterone levels and elevated cortisol, negatively impacting sperm production and quality [104]. On the other hand, a sedentary lifestyle contributes to obesity and poor metabolic health, both of which are linked to increased oxidative stress and inflammation. These factors can compromise meiotic division during spermatogenesis, increasing the likelihood of chromosomal segregation errors and thus the production of aneuploid sperm [105].

Heat exposure, particularly from sources like hot tubs, saunas, or even prolonged sitting with tight clothing or laptops on the lap, can elevate scrotal temperature beyond the optimal range for sperm production. The testes are highly sensitive to temperature, and sustained heat exposure disrupts the tightly regulated process of meiosis, damaging DNA and the spindle apparatus responsible for proper chromosomal separation [106]. Similarly, obesity contributes to elevated scrotal temperature due to increased fat deposition in the pelvic region, further compounding the risk. Moreover, obesity is associated with altered levels of reproductive hormones, such as decreased testosterone and increased estrogen, which impair spermatogenesis and can promote the generation of aneuploid gametes [107]. Together, these factors underline the importance of maintaining a healthy lifestyle, including appropriate physical activity, weight management, and temperature regulation, to safeguard genomic integrity in sperm [108].

## 6. Specific Chromosomal Syndromes Impacting Male Fertility

### 6.1. AZF Region Deletions

The AZF regions, that are located on the long arm of the Y chromosome (Yq11), are critical for spermatogenesis. Deletions in these regions, classified into AZFa, AZFb, and AZFc, are among the most common genetic causes of male infertility [109]. Each of these deletion affects spermatogenesis in a different way: AZFa deletions are typically associated with Sertoli cell-only syndrome [110], where no germ cells are present; AZFb deletions often result in a maturation arrest during meiosis; and AZFc deletions may cause varying degrees of oligozoospermia or complete azoospermia. While Assisted Reproductive Technologies (ART) may offer some options for AZFc-deleted individuals, the complete absence of sperm in AZFa and AZFb deletions generally results in very poor prognosis for biological fatherhood [111]. Moreover, these deletions are passed on to male offspring if sperm retrieval and ICSI (intracytoplasmic sperm injection) are used, raising ethical and genetic counseling concerns [112]. Genetic screening for AZF deletions is therefore a standard part of infertility workups, particularly in men with unexplained non-obstructive azoospermia or severe oligospermia. Early diagnosis can help guide fertility treatment decisions and prevent unnecessary interventions.

### 6.2. Kallmann Syndrome

Kallmann syndrome is a rare genetic condition characterized by the failure of Gonadotropin-Releasing Hormone (GnRH) neurons to migrate properly during embryonic development, leading to hypogonadotropic hypogonadism and anosmia (loss of smell). This hormonal deficiency results in underdeveloped secondary sexual characteristics and impaired spermatogenesis due to low levels of Luteinizing Hormone (LH) and Follicle-Stimulating Hormone (FSH) [55]. Most affected males present with delayed or absent puberty and infertility. However, fertility can often be restored through hormone replacement therapy using pulsatile GnRH or exogenous gonadotropins, which stimulate testicular function and sperm production [113]. The genetic causes of Kallmann syndrome are varied and complex. Variants of the several different genes, including *KAL1* (ANOS1 encodes the protein anosmin-1), *FGFR1* (Fibroblast Growth Factor Receptor 1), and *PROKR2* (Prokineticin Receptor 2), have been linked to the condition. Inheritance patterns can differ as well, with X-linked, autosomal dominant, and autosomal recessive forms all reported. Because of this genetic diversity, comprehensive genetic counseling is recommended for individuals and families affected by the condition. With early diagnosis and a personalized treatment plan, many patients with Kallmann syndrome can experience improved hormonal function, physical development, and even the possibility of achieving biological parenthood.

### 6.3. XX Male Syndrome (46,XX Testicular DSD)

XX male syndrome, also known as 46,XX testicular Disorder of Sex Development (DSD), is a rare condition in which individuals with a typically female chromosomal pattern (46,XX) develop male physical characteristics, often due to the presence of the Sex-determining Region Y protein (*SRY* gene)-normally found on the Y chromosome- translocated onto one of the X chromosomes [56]. Despite having a male phenotype, these individuals typically have small testes, azoospermia, and infertility due to the absence of other genes on the Y chromosome essential for spermatogenesis, including the AZF regions.

Clinically, most XX males have normal male genitalia at birth, but some may show signs of ambiguous genitalia or delayed puberty due to hormonal imbalances. Hormonal profiles often reveal hypergonadotropic hypogonadism, with elevated LH and FSH and low testosterone levels. Because of the absence of sperm production, biological paternity is generally not possible [114]. Management involves hormonal replacement if testosterone is deficient, and psychological support is often recommended due to gender identity and fertility implications [115].

## 7. Managing Chromosomal Risks in Male Infertility

Male fertility is uniquely sensitive to both genetic and modifiable risk factors, making intervention strategies a critical aspect of clinical management. Given the documented roles of oxidative stress, environmental toxins, poor diet, and advanced paternal age in promoting chromosomal instability, therapeutic approaches should aim to reduce cellular damage, support hormonal regulation, and improve meiotic fidelity. For example, minimizing occupational or environmental exposure to agents such as heavy metals, phthalates, and microplastics has been associated with lower levels of sperm DNA fragmentation and improved chromosomal segregation during meiosis [77,79,80,116]. Counseling regarding avoidance of heat exposure, smoking, and excessive alcohol intake also constitutes a practical and effective lifestyle intervention [83,84,85,104,105,106,107].

Nutritional optimization represents a promising and accessible therapeutic pathway. Supplementation with antioxidants—such as vitamins C, E, D, selenium, zinc, and coenzyme Q10—has been linked to improved sperm motility, morphology, and reduced aneuploidy rates in several studies [91,92,93,94,95,96,99,100,101]. These compounds help neutralize reactive oxygen species (ROS) that disrupt the spindle apparatus and damage chromatin, particularly in men with poor semen parameters. Additionally, micronutrients like folate and vitamin B12 play essential roles in DNA synthesis and methylation, further supporting chromosomal stability [91,92]. Despite encouraging data, larger randomized controlled trials are needed to validate their long-term effectiveness, particularly in subgroups with defined genetic risks or environmental exposures.

From a clinical perspective, genetic counseling and early screening should be integrated into routine fertility assessment, especially in cases of unexplained azoospermia, recurrent ART failure, or advanced paternal age. Men with identifiable chromosomal abnormalities (e.g., AZF deletions or Klinefelter syndrome) should receive tailored guidance on reproductive options and associated risks [12,13,111]. In select cases, micro-TESE combined with intracytoplasmic sperm injection (ICSI) offers a path to biological fatherhood, although the potential for passing on genetic defects must be addressed via preimplantation genetic testing (PGT) and ethical counseling [112,117]. As molecular understanding deepens, emerging therapeutic targets—such as cohesion stabilizers or spindle checkpoint modulators—may provide future avenues for correcting meiotic dysfunction at its source [41,54,69].

## 8. Discussion

Aneuploidy in male gametes represents a critical challenge in human reproduction, as it contributes to infertility, early pregnancy loss, and chromosomal syndromes in offspring. Although errors during female meiosis have been long recognized as a leading cause of aneuploid conceptions, recent evidence emphasizes the paternal contribution. Mechanisms underlying male aneuploidy include meiotic nondisjunction, spindle assembly checkpoint dysfunction, and abnormalities in the synaptonemal complex or cohesin proteins [32,36,41,118]. These issues disrupt the accurate separation of chromosomes during meiosis, often resulting in disomic or nullisomic sperm. Age-related degradation in these systems further exacerbates the risk of chromosomal missegregation in older men, increasing the prevalence of aneuploid sperm and subsequent syndromic conceptions such as Klinefelter or Down syndrome [40,61].

Environmental and lifestyle factors also significantly modulate spermatogenesis and genomic stability. Factors such as exposure to toxins (e.g., heavy metals, phthalates), oxidative stress, smoking, alcohol, and poor diet have all been implicated in disrupting meiotic processes. These influences may cause DNA damage, spindle defects, or hormonal imbalances, contributing to chromosomal missegregation [77,79,80,83,84,116]. Additionally, high body weight and frequent heat exposure can elevate testicular temperatures, which impairs sperm production and promotes aneuploidy [104,105,106,107]. On the other hand, moderate physical activity and antioxidant-rich diets offer a protective effect, mitigating the impact of oxidative stress and supporting meiotic fidelity. Nutrients such as folate, vitamin C, E, D, selenium, zinc, and coenzyme Q10 have been shown to enhance sperm quality and reduce DNA fragmentation rates [91,92,93,94,95,96,99,100,101]. Despite these insights, several important knowledge gaps persist. Submicroscopic chromosomal rearrangements—such as cryptic deletions or duplications—may remain undetected with standard cytogenetic techniques yet still contribute to idiopathic infertility. The incorporation of high-throughput genomic tools such as array comparative genomic hybridization (aCGH) or whole-genome sequencing could significantly improve diagnostic resolution in patients with unexplained non-obstructive azoospermia or severe oligozoospermia [109,117].

Another underexplored area is the interaction between genetic predisposition and environmental exposures. While toxins, oxidative stress, and poor nutrition have been associated with meiotic disruption, little is known about how specific genetic polymorphisms or epigenetic profiles might increase individual susceptibility. Longitudinal studies investigating gene–environment interactions could help identify high-risk groups and guide personalized fertility preservation strategies [54,58,81].

Additionally, concerns remain regarding the transgenerational transmission of chromosomal anomalies through assisted reproductive technologies, especially in men with known AZF deletions, Robertsonian translocations, or aneuploid sperm. The use of intracytoplasmic sperm injection (ICSI) may bypass natural selection barriers, raising ethical and clinical questions [111,112,117]. Future research should evaluate the long-term health outcomes of offspring conceived via ART under such conditions and reinforce the importance of genetic counseling and preimplantation genetic testing when appropriate.

From a therapeutic perspective, although antioxidant therapy, hormone regulation, and lifestyle modification have demonstrated potential benefits, evidence from randomized controlled trials is still limited. Furthermore, emerging molecular studies suggest that biomarkers—such as specific miRNAs or cohesin-related gene variants—may help detect early meiotic errors and offer targets for novel treatments [41,43,51]. Advancing this field will require multidisciplinary collaborations among clinicians, molecular geneticists, and reproductive biologists.

In conclusion, tackling male infertility related to chromosomal abnormalities necessitates an integrated approach combining precision diagnostics, risk stratification, and individualized therapy. Bridging the existing knowledge gaps through high-resolution genomic profiling and mechanistic studies will be essential for improving outcomes in both natural and assisted reproduction.

### 8.1. Synthesis of Genetic, Environmental, and Epigenetic Factors

Overall, chromosomal abnormalities in male infertility arise from a complex interplay between intrinsic genetic factors, environmental insults, and epigenetic regulation. While variants of cohesin or synaptonemal complex genes directly impair meiotic progression, external influences such as oxidative stress and endocrine-disrupting chemicals modulate chromatin stability and gene expression. Additionally, emerging data indicate that age-related epigenetic drift and DNA methylation changes may sensitize spermatogenic cells to external stressors. This multifactorial framework highlights that male infertility cannot be fully understood by analyzing individual pathways in isolation; rather, it requires an integrated model that accounts for cross-talk between molecular, environmental, and regulatory networks. This perspective underscores the need for multidimensional diagnostic approaches and personalized fertility management.

### 8.2. Future Directions

Future research should move toward integrated, multi-omic profiling of infertile men to uncover latent chromosomal instability and gene–environment interactions. One promising area is the three-dimensional organization of chromatin in spermatocytes, which may regulate access to recombination hotspots and influence the fidelity of chromosome segregation [119]. Additionally, non-coding RNAs, such as testis-specific miRNAs, are emerging as regulators of meiotic progression and potential biomarkers of checkpoint dysfunction [120,121,122]. Another innovative direction involves the use of CRISPR/Cas9-based systems not only to model chromosomal rearrangements in vitro, but also to explore targeted correction strategies in preclinical settings [123]. These novel perspectives may eventually translate into earlier diagnosis, preventive interventions, or even gene-targeted therapies in select cases of male infertility.

## 9. Conclusions

In summary, chromosomal abnormalities in males, both structural and numerical, play a substantial role in the genesis of aneuploid sperm and male infertility. These abnormalities arise from intrinsic defects in meiotic processes—such as nondisjunction, spindle assembly checkpoint dysfunction, and cohesion loss—and are further influenced by extrinsic factors like environmental toxins, aging, heat exposure, poor nutrition, and oxidative stress. Specific genetic syndromes, including AZF deletions, Kallmann syndrome, and 46,XX testicular DSD, highlight how disruptions in chromosomal integrity and hormonal regulation lead to impaired spermatogenesis and infertility. Understanding these mechanisms is essential not only for accurate diagnosis and clinical management but also for anticipating reproductive risks and offering appropriate genetic counseling.

Looking forward, integrating cytogenetic and molecular diagnostics with lifestyle-based interventions offers a promising path toward more personalized fertility care. Advances in high-resolution genomic tools may reveal hidden causes of idiopathic infertility, while emerging research on gene–environment interactions and meiotic biomarkers could enable earlier detection and more effective prevention. As the use of assisted reproductive technologies expands, especially in genetically affected individuals, ethical considerations and long-term outcomes must be carefully evaluated. A multidisciplinary, preventive, and precision-based approach is crucial to improving reproductive outcomes and reducing the burden of heritable chromosomal disorders in future generations.

## Figures and Tables

**Table 1:** Chromosomal Abnormalities in Male Infertility.

Abbreviation	Cytogenetic Notation	Prevalence in Infertile Men	Clinical Outcomes
**Klinefelter Syndrome**	47,XXY	almost 3–4% [9,12]	Azoospermia, low testosterone, small testes
**Jacob’s Syndrome**	47,XYY	1:1000 [9,14]	Often normospermic, but some with oligospermia
**Turner Mosaicism**	45,X/46,XY	2–5 per 100,000 males [10,18]	Gonadal dysgenesis, infertility, tumor risk
**AZF Deletions**	Yq11 microdeletions	5–20% in NOA [13,24]	Sertoli-cell only syndrome, meiotic arrest
**Robertsonian Translocations**	rob(13;14), rob(14;21), etc.	1:800 general population [20,22]	Risk of unbalanced gametes, miscarriages
**Reciprocal Translocations**	Various	almost 1% in infertile men [22,23]	Meiotic arrest, risk of offspring abnormalities
**Pericentric Inversion**	inv(9)(p12q13)	0.25–3% [26,27]	Often benign; possible link to infertility or miscarriage

**Table 2:** Gene variants associated with male infertility and their clinical significance.

Gene	Molecular Function	Associated Pathology	References
***STAG3***	Cohesin complex	Meiotic arrest, azoospermia	[41,43]
***SMC1β***	Sister chromatid cohesion	Recombination failure, meiotic defects	[42]
***SYCP3***	Synaptonemal complex	Meiotic failure, non-obstructive azoospermia	[51,53]
***SYCE1–3, TEX12***	Central SC proteins	Abnormal synapsis, meiotic arrest	[46,48]
***KAL1 (ANOS1)***	GnRH neuron migration	Kallmann syndrome, hypogonadism	[55]
***FGFR1***	FGF signaling	Hypogonadotropic hypogonadism	[55]
***SRY* (translocated)**	Sex determination	46,XX DSD, azoospermia	[56]

## References

[1] McFadden D.E., Friedman J.M. (1997). Chromosome Abnormalities in Human Beings. Mutat. Res. Mol. Mech. Mutagen..

[2] Jackson M., Marks L., May G.H.W., Wilson J.B. (2018). The Genetic Basis of Disease. Essays Biochem..

[3] Hassold T., Hall H., Hunt P. (2007). The Origin of Human Aneuploidy: Where We Have Been, Where We Are Going. Hum. Mol. Genet..

[4] Lo J.O., Feist C.D., Norton M.E., Caughey A.B. (2014). Noninvasive Prenatal Testing. Obstet. Gynecol. Surv..

[5] Hixson L., Goel S., Schuber P., Faltas V., Lee J., Narayakkadan A., Leung H., Osborne J. (2015). An Overview on Prenatal Screening for Chromosomal Aberrations. SLAS Technol..

[6] Natarajan A.T., Boei J.J.W.A. (2003). Formation of Chromosome Aberrations: Insights from FISH. Mutat. Res. Mutat. Res..

[7] Harton G.L., Tempest H.G. (2012). Chromosomal Disorders and Male Infertility. Asian J. Androl..

[8] Lamb D.J. (2025). Chromosome Defects and Male Factor Infertility. Fertil. Steril..

[9] Van Rijn S. (2019). A Review of Neurocognitive Functioning and Risk for Psychopathology in Sex Chromosome Trisomy (47,XXY, 47,XXX, 47, XYY). Curr. Opin. Psychiatry.

[10] Stochholm K., Holmgård C., Davis S.M., Gravholt C.H., Berglund A. (2024). Incidence, Prevalence, Age at Diagnosis, and Mortality in Individuals with 45,X/46,XY Mosaicism: A Population-Based Registry Study. Genet. Med..

[11] Maiburg M., Repping S., Giltay J. (2012). The Genetic Origin of Klinefelter Syndrome and Its Effect on Spermatogenesis. Fertil. Steril..

[12] Elahwany A., Elrefaey F.A., Alahwany H., Torad H., GamalEl Din S.F., Dawood R.M.S., Ragab M.W., Megawer A.F. (2025). Evaluation of the Predictors of Successful Sperm Retrieval of Micro-TESE in Cases with Mosaic Klinefelter versus Cases with Non-Mosaic Klinefelter: A Prospective Case Series Study. Basic Clin. Androl..

[13] Fesahat F., Montazeri F., Hoseini S.M. (2020). Preimplantation Genetic Testing in Assisted Reproduction Technology. J. Gynecol. Obstet. Hum. Reprod..

[14] Cannarella R., Pedano A., Compagnone M., La Vignera S., Condorelli R.A., Calogero A.E. (2025). Gonadal Function in Patients with 47,XYY Syndrome: A Systematic Review and Meta-Analysis. Endocr. Connect..

[15] Urbach A., Benvenisty N. (2009). Studying Early Lethality of 45,XO (Turner’s Syndrome) Embryos Using Human Embryonic Stem Cells. PLoS ONE.

[16] Ding F., Xu J., Xiong J., Li Q., Cheng Z., Deng L. (2025). Epidemiological Analysis of Turner Syndrome in Children Aged 0–14 Years: Global, Regional, and National Perspectives (1990–2021). Front. Endocrinol..

[17] Berglund A., Chang S., Lind-Holst M., Stochholm K., Gravholt C.H. (2025). The Epidemiology of Disorders of Sex Development. Best Pract. Res. Clin. Endocrinol. Metab..

[18] Corona L.E., Lee V.S., Weisman A.G., Rosoklija I., Hirsch J., Whitehead J., Almaghraby A., Papadakis J., Yuodsnukis B., Chen D. (2024). Mixed Gonadal Dysgenesis: A Narrative Literature Review and Clinical Primer for the Urologist. J. Urol..

[19] Lu L., Luo F., Wang X. (2022). Gonadal Tumor Risk in Pediatric and Adolescent Phenotypic Females with Disorders of Sex Development and Y Chromosomal Constitution with Different Genetic Etiologies. Front. Pediatr..

[20] Poot M., Hochstenbach R. (2021). Prevalence and Phenotypic Impact of Robertsonian Translocations. Mol. Syndromol..

[21] Gomes De Lima L., Guarracino A., Koren S., Potapova T., McKinney S., Rhie A., Solar S.J., Seidel C., Fagen B., Walenz B.P. (2024). The Formation and Propagation of Human Robertsonian Chromosomes. bioRxiv.

[22] Trieu S., Pham M., Le H., Vo H., Nguyen P., Tran T., Nguyen N., Trinh S. (2025). Survey of Structural Autosomal Abnormalities and Autosomal Variants in Infertile Patients Treated at Some IVF Centers in Vietnam. Appl. Clin. Genet..

[23] Zhang Z., Chen J., Zhang L., Wei R., Liu Z., Zhao D., Bi X., Liang L., Zhang X., Su D. (2025). Influence of the Sex of Translocation Carrier on Clinical Outcomes of Couples Undergoing Preimplantation Genetic Testing. Mol. Genet. Genom. Med..

[24] Rabinowitz M.J., Huffman P.J., Haney N.M., Kohn T.P. (2021). Y-Chromosome Microdeletions: A Review of Prevalence, Screening, and Clinical Considerations. Appl. Clin. Genet..

[25] Aoki S., Takeshima T., Mimura N., Seki H., Yumura Y. (2025). Successful Sperm Retrieval by Microdissection Testicular Sperm Extraction in a Man with Partial AZFb Deletion: A Case Report. Transl. Androl. Urol..

[26] Muthuvel A., Ravindran M., Chander A., Subbian C. (2016). Pericentric Inversion of Chromosome 9 Causing Infertility and Subsequent Successful in Vitro Fertilization. Niger. Med. J..

[27] Ting N.-S., Chen Y.-H., Chen S.-F., Chen P.-C. (2022). Successful Live Twin Birth through IVF/ICSI from a Couple with an Infertile Father with Pericentric Inversion of Chromosome 9 (P12q13): A Case with a High Aneuploidy Rate. Medicina.

[28] Mohsen-Pour N., Talebi T., Naderi N., Moghadam M.H., Maleki M., Kalayinia S. (2022). Chromosome 9 Inversion: Pathogenic or Benign?A Comprehensive Systematic Review of All Clinical Reports. Curr. Mol. Med..

[29] Mottola F., Santonastaso M., Ronga V., Finelli R., Rocco L. (2023). Polymorphic Rearrangements of Human Chromosome 9 and Male Infertility: New Evidence and Impact on Spermatogenesis. Biomolecules.

[30] Yuan J., Jin L., Wang M., Wei S., Zhu G., Xu B. (2023). Detection of Chromosome Aberrations in 17 054 Individuals with Fertility Problems and Their Subsequent Assisted Reproductive Technology Treatments in Central China. Hum. Reprod..

[31] Akalin H., Sahin I.O., Paskal S.A., Tan B., Yalcinkaya E., Demir M., Yakubi M., Caliskan B.O., Ekinci O.G., Ercan M. (2024). Evaluation of Chromosomal Abnormalities in the Postnatal Cohort: A Single-center Study on 14,242 Patients. J. Clin. Lab. Anal..

[32] Egozcue S. (2000). Human Male Infertility: Chromosome Anomalies, Meiotic Disorders, Abnormal Spermatozoa and Recurrent Abortion. Hum. Reprod. Update.

[33] Keen C., Hunter J.E., Allen E.G., Rocheleau C., Waters M., Sherman S.L. (2020). The Association between Maternal Occupation and down Syndrome: A Report from the National Down Syndrome Project. Int. J. Hyg. Environ. Health.

[34] Kocaaga A., Salik E.A. (2025). Chromosomal Abnormalities of Embryos from Sporadic and Recurrent Miscarriages: A Tertiary Center Experience. Mol. Biol. Rep..

[35] Pan Y., Chen C., Li H. (2025). Maternal Age-Related Gender Bias in Trisomy 21 and Trisomy 18. Birth Defects Res..

[36] Li Z., Liu Y., Jones A.W., Watanabe Y. (2024). Acetylation of Rec8 Cohesin Complexes Regulates Reductional Chromosome Segregation in Meiosis. Life Sci. Alliance.

[37] Chavda A.P., Ang K., Ivanov D. (2017). The Torments of the Cohesin Ring. Nucleus.

[38] Kulemzina I., Ang K., Zhao X., Teh J.-T., Verma V., Suranthran S., Chavda A.P., Huber R.G., Eisenhaber B., Eisenhaber F. (2016). A Reversible Association between Smc Coiled Coils Is Regulated by Lysine Acetylation and Is Required for Cohesin Association with the DNA. Mol. Cell.

[39] Sasaki M., Miyoshi N., Fujino S., Saso K., Ogino T., Takahashi H., Uemura M., Yamamoto H., Matsuda C., Yasui M. (2021). The Meiosis-Specific Cohesin Component Stromal Antigen 3 Promotes Cell Migration and Chemotherapeutic Resistance in Colorectal Cancer. Cancer Lett..

[40] Lee J. (2013). Roles of Cohesin and Condensin in Chromosome Dynamics During Mammalian Meiosis. J. Reprod. Dev..

[41] Van Der Bijl N., Röpke A., Biswas U., Wöste M., Jessberger R., Kliesch S., Friedrich C., Tüttelmann F. (2019). Mutations in the Stromal Antigen 3 (STAG3) Gene Cause Male Infertility Due to Meiotic Arrest. Hum. Reprod..

[42] Revenkova E., Eijpe M., Heyting C., Hodges C.A., Hunt P.A., Liebe B., Scherthan H., Jessberger R. (2004). Cohesin SMC1β Is Required for Meiotic Chromosome Dynamics, Sister Chromatid Cohesion and DNA Recombination. Nat. Cell Biol..

[43] Tsabai P.N., Pavlova N.S., Shatylko T.V., Kumykova Z.K., Stupko O.K., Kochetkova T.O., Lobanova N.N., Goltsov A.Y., Leukhina O.O., Shubina J. (2025). Novel STAG3 Variant Causes Oligoasthenoteratozoospermia with High Sperm Aneuploidy Rate. J. Assist. Reprod. Genet..

[44] Liu W., Gao X., Zhang H., Liu R., Cao Y., Yu R., Fang G., Ma J., Zhao S. (2021). Analysis of STAG3 Variants in Chinese Non-Obstructive Azoospermia Patients with Germ Cell Maturation Arrest. Sci. Rep..

[45] Liu C., Zhang Y., Zhao Y., Luo H. (2025). A Novel Loss-of-Function SYCP2 Variant Causes Asthenoteratozoospermia in Infertile Males. Front. Genet..

[46] Heyting C. (1996). Synaptonemal Complexes: Structure and Function. Curr. Opin. Cell Biol..

[47] Billmyre K.K., Kesler E.A., Tsuchiya D., Corbin T.J., Weaver K., Moran A., Yu Z., Adams L., Delventhal K., Durnin M. (2023). SYCP1 Head-to-Head Assembly Is Required for Chromosome Synapsis in Mouse Meiosis. Sci. Adv..

[48] Zwettler F.U., Spindler M.-C., Reinhard S., Klein T., Kurz A., Benavente R., Sauer M. (2020). Tracking down the Molecular Architecture of the Synaptonemal Complex by Expansion Microscopy. Nat. Commun..

[49] Gray J.E., Schenker M., Nahit Şendur M.A., Leonova V., Kowalski D., Kato T., Orlova R., Chih-Hsin Yang J., Langleben A., Pilz A. (2024). The Phase 3 KEYLYNK-006 Study of Pembrolizumab plus Olaparib versus Pembrolizumab plus Pemetrexed as Maintenance Therapy for Metastatic Nonsquamous Non–Small-Cell Lung Cancer. J. Thorac. Oncol..

[50] Enguita-Marruedo A., Van Cappellen W.A., Hoogerbrugge J.W., Carofiglio F., Wassenaar E., Slotman J.A., Houtsmuller A., Baarends W.M. (2018). Live Cell Analyses of Synaptonemal Complex Dynamics and Chromosome Movements in Cultured Mouse Testis Tubules and Embryonic Ovaries. Chromosoma.

[51] Stouffs K., Vandermaelen D., Tournaye H., Liebaers I., Lissens W. (2011). Mutation Analysis of Three Genes in Patients with Maturation Arrest of Spermatogenesis and Couples with Recurrent Miscarriages. Reprod. Biomed. Online.

[52] Yuan L., Liu J.-G., Zhao J., Brundell E., Daneholt B., Höög C. (2000). The Murine SCP3 Gene Is Required for Synaptonemal Complex Assembly, Chromosome Synapsis, and Male Fertility. Mol. Cell.

[53] Miyamoto T., Minase G., Shin T., Ueda H., Okada H., Sengoku K. (2017). Human Male Infertility and Its Genetic Causes. Reprod. Med. Biol..

[54] Zhou G., Zhang M., Zhang J., Feng Y., Xie Z., Liu S., Zhu D., Luo Y. (2022). The Gene Regulatory Role of Non-Coding RNAs in Non-Obstructive Azoospermia. Front. Endocrinol..

[55] Rohayem J., Zitzmann M., Nieschlag E. (2015). Congenital Hypogonadotropic Hypogonadism and Kallmann’s Syndrome★. Reference Module in Biomedical Sciences.

[56] Li T.-F., Wu Q.-Y., Zhang C., Li W.-W., Zhou Q., Jiang W.-J., Cui Y.-X., Xia X.-Y., Shi Y.-C. (2014). 46,XX Testicular Disorder of Sexual Development with SRY-Negative Caused by Some Unidentified Mechanisms: A Case Report and Review of the Literature. BMC Urol..

[57] Du Fossé N.A., Van Der Hoorn M.-L.P., Van Lith J.M.M., Le Cessie S., Lashley E.E.L.O. (2020). Advanced Paternal Age Is Associated with an Increased Risk of Spontaneous Miscarriage: A Systematic Review and Meta-Analysis. Hum. Reprod. Update.

[58] Kaltsas A., Markou E., Kyrgiafini M.-A., Zikopoulos A., Symeonidis E.N., Dimitriadis F., Zachariou A., Sofikitis N., Chrisofos M. (2025). Oxidative-Stress-Mediated Epigenetic Dysregulation in Spermatogenesis: Implications for Male Infertility and Offspring Health. Genes.

[59] Kuchakulla M., Narasimman M., Khodamoradi K., Khosravizadeh Z., Ramasamy R. (2021). How Defective Spermatogenesis Affects Sperm DNA Integrity. Andrologia.

[60] Colaco S., Sakkas D. (2018). Paternal Factors Contributing to Embryo Quality. J. Assist. Reprod. Genet..

[61] Dviri M., Madjunkova S., Koziarz A., Madjunkov M., Mashiach J., Nekolaichuk E., Trivodaliev K., Al-Asmar N., Moskovtsev S.I., Librach C. (2021). Is There an Association between Paternal Age and Aneuploidy? Evidence from Young Donor Oocyte-Derived Embryos: A Systematic Review and Individual Patient Data Meta-Analysis. Hum. Reprod. Update.

[62] Eaker S., Pyle A., Cobb J., Handel M.A. (2001). Evidence for Meiotic Spindle Checkpoint from Analysis of Spermatocytes from Robertsonian-Chromosome Heterozygous Mice. J. Cell Sci..

[63] Shannon K.B., Canman J.C., Salmon E.D. (2002). Mad2 and BubR1 Function in a Single Checkpoint Pathway That Responds to a Loss of Tension. Mol. Biol. Cell.

[64] Marchetti F., Venkatachalam S. (2010). The Multiple Roles of Bub1 in Chromosome Segregation during Mitosis and Meiosis. Cell Cycle.

[65] Mihajlović A.I., Byers C., Reinholdt L., FitzHarris G. (2023). Spindle Assembly Checkpoint Insensitivity Allows MEIOSIS-II despite Chromosomal Defects in Aged Eggs. EMBO Rep..

[66] Vogt E., Kirsch-Volders M., Parry J., Eichenlaub-Ritter U. (2008). Spindle Formation, Chromosome Segregation and the Spindle Checkpoint in Mammalian Oocytes and Susceptibility to Meiotic Error. Mutat. Res. Toxicol. Environ. Mutagen..

[67] Ribagorda M., Berríos S., Solano E., Ayarza E., Martín-Ruiz M., Gil-Fernández A., Parra M.T., Viera A., Rufas J.S., Capanna E. (2019). Meiotic Behavior of a Complex Hexavalent in Heterozygous Mice for Robertsonian Translocations: Insights for Synapsis Dynamics. Chromosoma.

[68] Xie C., Wang W., Tu C., Meng L., Lu G., Lin G., Lu L.-Y., Tan Y.-Q. (2022). Meiotic Recombination: Insights into Its Mechanisms and Its Role in Human Reproduction with a Special Focus on Non-Obstructive Azoospermia. Hum. Reprod. Update.

[69] Wieland J., Buchan S., Sen Gupta S., Mantzouratou A. (2022). Genomic Instability and the Link to Infertility: A Focus on Microsatellites and Genomic Instability Syndromes. Eur. J. Obstet. Gynecol. Reprod. Biol..

[70] Zhang C., Guo Y., Yang Y., Du Z., Fan Y., Zhao Y., Yuan S. (2023). Oxidative Stress on Vessels at the Maternal-Fetal Interface for Female Reproductive System Disorders: Update. Front. Endocrinol..

[71] Potapova T., Gorbsky G. (2017). The Consequences of Chromosome Segregation Errors in Mitosis and Meiosis. Biology.

[72] Kops G.J.P.L., Snel B., Tromer E.C. (2020). Evolutionary Dynamics of the Spindle Assembly Checkpoint in Eukaryotes. Curr. Biol..

[73] Keefe D.L. (2020). Telomeres and Genomic Instability during Early Development. Eur. J. Med. Genet..

[74] Rosenbusch B., Sterzik K. (1994). Cytogenetics of Human Spermatozoa. The Frequency of Various Chromosome Aberrations and Their Relationship to Clinical and Biological Parameters. Arch. Gynecol. Obstet..

[75] Hwang K., Weedin J.W., Lamb D.J. (2010). The Use of Fluorescent *in Situ* Hybridization in Male Infertility. Ther. Adv. Urol..

[76] Chamayou S., Giacone F., Cannarella R., Guglielmino A. (2023). What Does Intracytoplasmic Sperm Injection Change in Embryonic Development? The Spermatozoon Contribution. J. Clin. Med..

[77] Ramos-Treviño J., Bassol-Mayagoitia S., Hernández-Ibarra J.A., Ruiz-Flores P., Nava-Hernández M.P. (2018). Toxic Effect of Cadmium, Lead, and Arsenic on the Sertoli Cell: Mechanisms of Damage Involved. DNA Cell Biol..

[78] Peng Y., Zhang W., Chen Y., Zhang L., Shen H., Wang Z., Tian S., Yang X., Cui D., He Y. (2023). Engineering C-Met-CAR NK-92 Cells as a Promising Therapeutic Candidate for Lung Adenocarcinoma. Pharmacol. Res..

[79] Asadi N. (2017). The Impact of Oxidative Stress on Testicular Function and the Role of Antioxidants in Improving It: A Review. J. Clin. Diagn. Res..

[80] Daniels D., Berger Eberhardt A. (2024). Climate Change, Microplastics, and Male Infertility. Curr. Opin. Urol..

[81] Estill M.S., Krawetz S.A. (2016). The Epigenetic Consequences of Paternal Exposure to Environmental Contaminants and Reproductive Toxicants. Curr. Environ. Health Rep..

[82] Xu L., Chen S., Fu W., Lin X., Zhang F., Qin G., Yuan Z., Huang B. (2025). Environmental Toxicant 2,3,7,8-Tetrachlorodibenzo-p-Dioxin Induces Non-Obstructive Azoospermia: New Insights from Network Toxicology, Integrated Machine Learning, and Biomolecular Modeling. Ecotoxicol. Environ. Saf..

[83] Firouzabadi A.M., Henkel R., Tofighi Niaki M., Fesahat F. (2025). Adverse Effects of Nicotine on Human Sperm Nuclear Proteins. World J. Mens Health.

[84] Tommasi S., Kitapci T.H., Blumenfeld H., Besaratinia A. (2022). Secondhand Smoke Affects Reproductive Functions by Altering the Mouse Testis Transcriptome, and Leads to Select Intron Retention in Pde1a. Environ. Int..

[85] Nguyen-Thanh T., Hoang-Thi A.-P., Anh Thu D.T. (2023). Investigating the Association between Alcohol Intake and Male Reproductive Function: A Current Meta-Analysis. Heliyon.

[86] Lim J., Squire E., Jung K.-M. (2023). Phytocannabinoids, the Endocannabinoid System and Male Reproduction. World J. Mens Health.

[87] Schifano N., Chiappini S., Mosca A., Miuli A., Santovito M.C., Pettorruso M., Capogrosso P., Dehò F., Martinotti G., Schifano F. (2022). Recreational Drug Misuse and Its Potential Contribution to Male Fertility Levels’ Decline: A Narrative Review. Brain Sci..

[88] Smith S.J., Lopresti A.L., Fairchild T.J. (2023). The Effects of Alcohol on Testosterone Synthesis in Men: A Review. Expert Rev. Endocrinol. Metab..

[89] Koh K., Kim S.S., Kim J.-S., Jung J.-G., Yoon S.-J., Suh W.Y., Kim H.G., Kim N. (2022). Relationship between Alcohol Consumption and Testosterone Deficiency According to Facial Flushes among Middle-Aged and Older Korean Men. Korean J. Fam. Med..

[90] Pascoal G.D.F.L., Geraldi M.V., Maróstica M.R., Ong T.P. (2022). Effect of Paternal Diet on Spermatogenesis and Offspring Health: Focus on Epigenetics and Interventions with Food Bioactive Compounds. Nutrients.

[91] Gaskins A.J., Chiu Y.-H., Williams P.L., Ford J.B., Toth T.L., Hauser R., Chavarro J.E. (2015). Association between Serum Folate and Vitamin B-12 and Outcomes of Assisted Reproductive Technologies. Am. J. Clin. Nutr..

[92] Young S.S., Eskenazi B., Marchetti F.M., Block G., Wyrobek A.J. (2008). The Association of Folate, Zinc and Antioxidant Intake with Sperm Aneuploidy in Healthy Non-Smoking Men. Hum. Reprod..

[93] Al-Azemi M., Omu A., Fatinikun T., Mannazhath N., Abraham S. (2009). Factors Contributing to Gender Differences in Serum Retinol and α-Tocopherol in Infertile Couples. Reprod. Biomed. Online.

[94] Colagar A.H., Marzony E.T. (2009). Ascorbic Acid in Human Seminal Plasma: Determination and Its Relationship to Sperm Quality. J. Clin. Biochem. Nutr..

[95] Matorras R., Pérez-Sanz J., Corcóstegui B., Pérez-Ruiz I., Malaina I., Quevedo S., Aspichueta F., Crisol L., Martinez-Indart L., Prieto B. (2020). Effect of Vitamin E Administered to Men in Infertile Couples on Sperm and Assisted Reproduction Outcomes: A Double-Blind Randomized Study. FS Rep..

[96] Keskes-Ammar L., Feki-Chakroun N., Rebai T., Sahnoun Z., Ghozzi H., Hammami S., Zghal K., Fki H., Damak J., Bahloul A. (2003). Sperm Oxidative Stress and the Effect of an Oral Vitamin E And Selenium Supplement on Semen Quality in Infertile Men. Arch. Androl..

[97] Kinuta K., Tanaka H., Moriwake T., Aya K., Kato S., Seino Y. (2000). Vitamin D Is an Important Factor in Estrogen Biosynthesis of Both Female and Male Gonads*. Endocrinology.

[98] Blomberg Jensen M., Bjerrum P.J., Jessen T.E., Nielsen J.E., Joensen U.N., Olesen I.A., Petersen J.H., Juul A., Dissing S., Jørgensen N. (2011). Vitamin D Is Positively Associated with Sperm Motility and Increases Intracellular Calcium in Human Spermatozoa. Hum. Reprod..

[99] Zhao J., Dong X., Hu X., Long Z., Wang L., Liu Q., Sun B., Wang Q., Wu Q., Li L. (2016). Zinc Levels in Seminal Plasma and Their Correlation with Male Infertility: A Systematic Review and Meta-Analysis. Sci. Rep..

[100] Nadjarzadeh A., Sadeghi M.R., Amirjannati N., Vafa M.R., Motevalian S.A., Gohari M.R., Akhondi M.A., Yavari P., Shidfar F. (2011). Coenzyme Q10 Improves Seminal Oxidative Defense but Does Not Affect on Semen Parameters in Idiopathic Oligoasthenoteratozoospermia: A Randomized Double-Blind, Placebo Controlled Trial. J. Endocrinol. Investig..

[101] Balercia G., Buldreghini E., Vignini A., Tiano L., Paggi F., Amoroso S., Ricciardo-Lamonica G., Boscaro M., Lenzi A., Littarru G. (2009). Coenzyme Q10 Treatment in Infertile Men with Idiopathic Asthenozoospermia: A Placebo-Controlled, Double-Blind Randomized Trial. Fertil. Steril..

[102] Zańko A., Pawłowski M., Milewski R. (2025). The Impact of Physical Exercise on Male Fertility Through Its Association with Various Processes and Aspects of Human Biology. J. Clin. Med..

[103] Antinozzi C., Di Luigi L., Sireno L., Caporossi D., Dimauro I., Sgrò P. (2025). Protective Role of Physical Activity and Antioxidant Systems During Spermatogenesis. Biomolecules.

[104] Cadegiani F.A., Kater C.E. (2017). Hormonal Aspects of Overtraining Syndrome: A Systematic Review. BMC Sports Sci. Med. Rehabil..

[105] Mennitti C., Farina G., Imperatore A., De Fonzo G., Gentile A., La Civita E., Carbone G., De Simone R.R., Di Iorio M.R., Tinto N. (2024). How Does Physical Activity Modulate Hormone Responses?. Biomolecules.

[106] Hoang-Thi A.-P., Dang-Thi A.-T., Phan-Van S., Nguyen-Ba T., Truong-Thi P.-L., Le-Minh T., Nguyen-Vu Q.-H., Nguyen-Thanh T. (2022). The Impact of High Ambient Temperature on Human Sperm Parameters: A Meta-Analysis. Iran. J. Public Health.

[107] Barbagallo F., Condorelli R.A., Mongioì L.M., Cannarella R., Cimino L., Magagnini M.C., Crafa A., La Vignera S., Calogero A.E. (2021). Molecular Mechanisms Underlying the Relationship between Obesity and Male Infertility. Metabolites.

[108] Sharma R., Biedenharn K.R., Fedor J.M., Agarwal A. (2013). Lifestyle Factors and Reproductive Health: Taking Control of Your Fertility. Reprod. Biol. Endocrinol..

[109] Koh E., Sin H., Fukushima M., Namiki M. (2010). Azoospermia Factor and Male Infertility. Reprod. Med. Biol..

[110] Wang X., Liu X., Qu M., Li H. (2023). Sertoli Cell-Only Syndrome: Advances, Challenges, and Perspectives in Genetics and Mechanisms. Cell. Mol. Life Sci..

[111] Colaco S., Modi D. (2018). Genetics of the Human Y Chromosome and Its Association with Male Infertility. Reprod. Biol. Endocrinol..

[112] Deng C.-Y., Zhang Z., Tang W.-H., Jiang H. (2023). Microdeletions and Vertical Transmission of the Y-Chromosome Azoospermia Factor Region. Asian J. Androl..

[113] Valdes-Socin H., Rubio Almanza M., TomÃ© FernÃ¡ndez-Ladreda M., Debray F.G., Bours V., Beckers A. (2014). Reproduction, Smell, and Neurodevelopmental Disorders: Genetic Defects in Different Hypogonadotropic Hypogonadal Syndromes. Front. Endocrinol..

[114] Wiese C.B., Avetisyan R., Reue K. (2023). The Impact of Chromosomal Sex on Cardiometabolic Health and Disease. Trends Endocrinol. Metab..

[115] Bouvattier C. (2010). Disorders of Sex Development. Pediatric Urology.

[116] Peng Y., He Q. (2024). Reproductive Toxicity and Related Mechanisms of Micro(Nano)Plastics in Terrestrial Mammals: Review of Current Evidence. Ecotoxicol. Environ. Saf..

[117] Martin R.H. (2008). Cytogenetic Determinants of Male Fertility. Hum. Reprod. Update.

[118] Sun S.-C., Kim N.-H. (2012). Spindle Assembly Checkpoint and Its Regulators in Meiosis. Hum. Reprod. Update.

[119] Zhang S., Tao W., Han J.-D.J. (2022). 3D Chromatin Structure Changes during Spermatogenesis and Oogenesis. Comput. Struct. Biotechnol. J..

[120] Chen X., Li X., Guo J., Zhang P., Zeng W. (2017). The Roles of microRNAs in Regulation of Mammalian Spermatogenesis. J. Anim. Sci. Biotechnol..

[121] Mobasheri M.B., Babatunde K.A. (2019). Testicular miRNAs in Relation to Spermatogenesis, Spermatogonial Stem Cells and Cancer/Testis Genes. Sci. Afr..

[122] Walker W.H. (2022). Regulation of Mammalian Spermatogenesis by miRNAs. Semin. Cell Dev. Biol..

[123] Lu Y., Oura S., Matsumura T., Oji A., Sakurai N., Fujihara Y., Shimada K., Miyata H., Tobita T., Noda T. (2019). CRISPR/Cas9-Mediated Genome Editing Reveals 30 Testis-Enriched Genes Dispensable for Male Fertility in Mice†. Biol. Reprod..

